# Vitamin D Levels Vary during Antiviral Treatment but Are Unable to Predict Treatment Outcome in HCV Genotype 1 Infected Patients

**DOI:** 10.1371/journal.pone.0087974

**Published:** 2014-02-07

**Authors:** Georgios Grammatikos, Christian Lange, Simone Susser, Susanne Schwendy, Nektarios Dikopoulos, Peter Buggisch, Jens Encke, Gerlinde Teuber, Tobias Goeser, Robert Thimme, Hartwig Klinker, Wulf O. Boecher, Ewert Schulte-Frohlinde, Marissa Penna-Martinez, Klaus Badenhoop, Stefan Zeuzem, Thomas Berg, Christoph Sarrazin

**Affiliations:** 1 J. W. Goethe University Hospital, Medizinische Klinik 1, Frankfurt am Main, Germany; 2 Uniklinik TU München, München, Germany; 3 Uniklinik Ulm, Ulm, Germany; 4 Uniklinik Hamburg, Hamburg, Germany; 5 Uniklinik Heidelberg, Heidelberg, Germany; 6 Interdisziplinäres Facharztzentrum, Frankfurt am Main, Germany; 7 Uniklinik Köln, Köln, Germany; 8 Uniklinik Freiburg, Freiburg, Germany; 9 Uniklinik Würzburg, Würzburg, Germany; 10 Uniklinik Mainz, Mainz, Germany; 11 Uniklinik Leipzig, Leipzig, Germany; Lady Davis Institute for Medical Research/McGill University, Canada

## Abstract

**Background:**

Different parameters have been determined for prediction of treatment outcome in hepatitis c virus genotype 1 infected patients undergoing pegylated interferon, ribavirin combination therapy. Results on the importance of vitamin D levels are conflicting. In the present study, a comprehensive analysis of vitamin D levels before and during therapy together with single nucleotide polymorphisms involved in vitamin D metabolism in the context of other known treatment predictors has been performed.

**Methods:**

In a well characterized prospective cohort of 398 genotype 1 infected patients treated with pegylated interferon-α and ribavirin for 24–72 weeks (INDIV-2 study) 25-OH-vitamin D levels and different single nucleotide polymorphisms were analyzed together with known biochemical parameters for a correlation with virologic treatment outcome.

**Results:**

Fluctuations of more than 5 (10) ng/ml in 25-OH-vitamin D-levels have been observed in 66 (39) % of patients during the course of antiviral therapy and neither pretreatment nor under treatment 25-OH-vitamin D-levels were associated with treatment outcome. The *DHCR7-TT-*polymorphism within the 7-dehydrocholesterol-reductase showed a significant association (*P* = 0.031) to sustained viral response in univariate analysis. Among numerous further parameters analyzed we found that age (OR = 1.028, CI = 1.002–1.056, *P* = 0.035), cholesterol (OR = 0.983, CI = 0.975–0.991, *P*<0.001), ferritin (OR = 1.002, CI = 1.000–1.004, *P* = 0.033), gGT (OR = 1.467, CI = 1.073–2.006, *P* = 0.016) and *IL28B*-genotype (OR = 2.442, CI = 1.271–4.695, *P* = 0.007) constituted the strongest predictors of treatment response.

**Conclusions:**

While 25-OH-vitamin D-levels levels show considerable variations during the long-lasting course of antiviral therapy they do not show any significant association to treatment outcome in genotype 1 infected patients.

## Introduction

Almost 3% of the world population is chronically infected with hepatitis c virus (HCV) and thus potentially confronted with life-threatening complications such as liver cirrhosis and liver cancer [1]. During permanent attempts to improve therapeutic strategies, beside the recent approval of two novel direct acting antiviral compounds [2], several predictors of treatment response have been identified [3] with recent studies including gamma-glutamyl-transferase (gGT) [4], cholesterol [5], early viral kinetics [6], interferon-γ-inducible-protein-10 (IP10) [7], ferritin [8] and the interleukin-28B (*IL28B*) polymorphism [9] as further important predictors of sustained viral response (SVR) as well. However, the pursuit of further surrogate factors able to optimize therapeutic regimes remains challenging.

Beside the above mentioned parameters many studies suggested vitamin D (VitD) as an additional predictor of SVR, whereas low pretreatment levels of 25-OH-Vitamin D_3_ (25(OH)D_3_) (<20 ng/ml) associated significantly with low responsiveness to antiviral therapy[10]–[14]. However, recent findings also indicate that the pretreatment concentration of VitD is not always capable of predicting treatment outcome in chronic HCV infection [15] as well as in HCV/HIV-coinfected patients [16], [17]. Moreover, serum concentrations of 25(OH)D_3_ are getting affected by various factors such as nutrition, comorbidities and seasonal sunlight exposure [18] and since an additional hydroxylation step is needed, 25(OH)D_3_-levels just offer an indirect association to the active form of VitD, 1,25(OH)_2_D_3._ The latter has a half-life of only 4 hours and is detectable in much lower serum concentrations than 25(OH)D_3_. 1,25(OH)_2_D_3_ is additionally strongly affected by the serum levels of calcium, phosphate and parathyroid hormone with clinical guidelines still recommending the routine assessment of 25(OH)D_3_ as the appropriate parameter in order to monitor the VitD status of patients [19]. In this context genetic polymorphisms within key enzymes regulating the pathophysiology of VitD have been shown to affect substantially VitD signaling in clinical diseases [20], [21]. In chronic HCV infection genetic polymorphisms within VitD binding proteins [12], the *CYP27B1*-hydroxylase [22] and the VitD-receptor [23] have been shown to correlate significantly with the outcome of antiviral therapy.

Purpose of the present study is therefore to evaluate the predictive potential of serum VitD levels both prior to as well as during antiviral therapy in a large (n = 398), well characterized cohort of genotype 1 HCV infected patients treated prospectively with pegylated interferon-α (PEG-IFNα) and ribavirin (RBV) for 24–72 weeks (INDIV-2 study) [24]. Since liver histology and genetic data were available in the majority of patients, we additionally evaluated genetic polymorphisms within major enzymes regulating 25(OH)D_3_- and 1,25(OH)_2_D_3_-concentrations, *CYP2R1*, *CYP27B1, DHCR7, CYP24A1* and within VitD binding proteins (*DBP)*. Findings concerning the prediction of treatment outcome were further analyzed in association with several prognostic parameters being available prior to initiation, during and after completion of antiviral therapy in our patient cohort.

## Patients and Methods

### Patients

In the current study we evaluated retrospectively the predictive potential of various parameters regarding treatment outcome in a cohort of 398 patients treated prospectively with PEG-IFNα/RBV as previously described [24]. Histologic results of liver biopsies were available for 378 out of 398 patients (95% of all patients). Genotyping for *IL28B*, *CYP27B1*, *CYP2R1, CYP24A1, DHCR7* and *DBP* was determined in all patients being agreeable to genetic analysis (375 out of 398, 94% of all patients). Demographic characteristics and clinical parameters assessed prior to, during and after antiviral therapy were extracted from the INDIV-2 clinical database [24]. Uni- and multivariate analysis were performed to evaluate possible associations between SVR and various variables such as age, sex, body mass index (BMI), liver fibrosis, *IL28B*-, *CYP27B1*-, *CYP2R1-, CYP24A1-, DHCR7-, DBP*-genotype, VitD levels at baseline and week 24 of antiviral therapy, IP10-levels at baseline and week 1 and 4 of antiviral therapy, alanine aminotransferase (ALT), aspartate aminotransferase (AST), gamma-glutamyl-transferase (gGT), alkaline phosphatase (AP), bilirubin, cholesterol, triglycerides, thyroid stimulating hormone (TSH), ferritin and homeostatic model assessment of insulin resistance (HOMA) by means of a logistic regression model with SVR being the dependent variable as explained further in the statistical analysis. Clinical biochemistry values such as glucose, cholesterol and triglycerides were transformed to uniform measurement units to render data comparable to each other. The design of the study has been described previously [24]. The study was performed in accordance with the Declaration of Helsinki and was approved by the local ethics committee (Ethik-Kommission, University Hospital Frankfurt). All patients had signed a written informed consent prior to study inclusion.

### Quantification of IP10- and VitD-levels

The commercially available human CXCL10/IP-10 Quantikine ELISA Kit, provided by R&D systems, was used for quantification of IP10 levels. 75 µl patient-serum was used for every measurement performed according to the manufacturer’s instructions. 25(OH)D_3_ concentrations were quantified by the I^125^–radioimmunoassay IA Kit, provided by DiaSorin. In this case 50 µl patient-serum was used for every measurement performed according to the manufacturer’s instructions. Quantification of both IP10- and 25(OH)D_3_- levels and genotyping of VitD genetic polymorphisms was performed retrospectively whereas all samples were stored at −80°C until assayed.

### Genotyping of Single Nucleotide Polymorphisms

We evaluated the predictive potential of *IL28B* (rs12979860) variants, *CYP27B1* (rs10877012) promoter-, the *CYP2R1* (rs10741657) hydroxylase-, the *CYP24A1* (rs6013897) hydroxylase-, the DHCR7 (rs12785878 and rs7944926) reductase- as well as *DBP* (rs2282679, rs4588 and rs7041) binding protein-polymorphisms on treatment outcome. Genotyping of *IL28B* was performed as described before [25]. For the determination of *CYP2R1-* (rs10741657), *CYP27B1*-(rs10877012) and *DBP* (rs4588 and rs7041) polymorphisms DNA was amplified with the respective primers, with PCR conditions and enzymes as previously described [26], [27]. The polymorphisms within the DHCR7 reductase-gene (rs12785878/C_32063037_10 and rs7944926/C_12043682_10), *DBP* (rs2282679/C_26407519_10) and *CYP24A1*- (rs6013897/C_22958084_10) were analyzed in the laboratory of molecular endocrinology of our department using Taqman assays in an ABI 7300 PCR System under the conditions recommended by the manufacturer (Applied Biosystems, Darmstadt, Germany) as previously described [26].

### Statistical Analysis

Statistical analysis of the data was performed using the BIAS software package (BiAS for Windows, program version 9 ©, Epsilon, 1989–2012). Continuous variables are presented as mean values (range) and categorical variables are presented as frequencies (%). Assessment of significant associations between continuous variables as for instance age, BMI, ALT, AST, gGT, AP, bilirubin, ferritin, HOMA-index, cholesterol, triglycerides, 25(OH)D_3_, IP10, TSH and treatment endpoints (SVR) were carried out via the Wilcoxon-Mann-Whitney-U-test. Dichotomic variables (e.g. single-nucleotide-polymorphisms (SNP’s), fibrosis stage, sex, treatment outcome) were assessed by means of contingency tables, Mantel-Haenszel-test, as appropriate. After univariate analysis stepwise logistic regression analysis with a backward selection, using a P value ≥0.1 for removal from the model, was performed to identify independent predictors of SVR. Parameters with significant associations derived from univariate analysis as well as sex and age were included into the model. Only patients with complete data for the predicting covariates were included in multivariate analysis. P values <0.05 were considered to be significant.

## Results

### Patient Characteristics

The INDIV-2-study cohort included 398 patients with genotype-1 chronic HCV infection treated prospectively with PEG-IFNα/RBV for different treatment durations. Length of treatment was individualized for every patient according to baseline viral load and virologic response rates assessed at different time points upon initiation of antiviral therapy [24]. Biochemical, virologic, histologic and demographic characteristics of the patient cohort are shown in Table 1.

**Table 1:** Patient demographic, biochemical and genetic characteristics.

Patient demographic biochemical and genetic data	Mean (Range)
**Age**	43,1 (18–70)
Sex (female/male) *n, [%]	182* [45,7%]/216* [54,3%]
**BMI (kg/m^2^)**	25,5 (16,6–45,5)
**ALT_fact (times upper limit of normal, IU/l)**	1,9 (0,3–12,9)
**AST_fact (times upper limit of normal, IU/l)**	1,34 (0,43–7,96)
**GGT_fact (times upper limit of normal, IU/l)**	1,3 (0,2–16,1)
**HOMA-index**	3,19 (0,1–22,6)
**Ferritin (µg/l)**	174 (3,7–1846)
**Triglyceride (mg/dl)**	104,4 (16–490)
**AP (IU/l)**	70,4 (32–204)
**TSH (µIU/ml)**	1,52 (0,01–12)
**Fasting glucose (mg/dl)**	94 (60–201)
**Cholesterol (mg/dl)**	174 (61–320)
**Vitamin D baseline (ng/ml)**	18,7 (3–84,3)
**Vitamin D TW 24 (ng/ml)**	19,5 (3,2–61,9)
**IP10 baseline (pg/ml)**	254 (41,2–2000)
**HCV-RNA (log IU/ml)**	5,6 (2,8–6,9)
**HCV-subtype (1a/1b/1c)**	109 (33,3%)/210 (64,2%)/8 (2,4%)
***IL28B-rs12979860*** ** (CC/non_CC)**	117 (31,8%)/250 (68,1%)
***CYP27B1-rs10877012*** ** (AA/non_AA)**	39 (10,6%)/327 (89,3%)
***CYP2R1- rs10741657*** ** (AA/non_AA)**	51 (13,9%)/315 (86,1%)
***DHCR7-rs12785878*** ** (TT/non_TT)**	198 (52,8%)/177 (47,2%)
***CYP24A1-rs6013897*** ** (TT/non_TT)**	232 (61,8%)/143 (38,1%)
***DHCR7-rs7944926*** ** (GG/non_GG)**	204 (54,2%)/172 (45,7%)
***DBP-rs2282679*** ** (TT/non_TT)**	196 (52,2%)/179 (47,7%)
***DBP-rs4588*** ** (CC/non_CC)**	188 (50,1%)/187 (49,9%)
***DBP-rs7041*** ** (GG/non_GG)**	115 (30,6%)/260 (69,3%)
Fibrosis stage (0–2/3–4) *n, [%]	321 [84,9%]/57 [15,1%]

A VitD deficiency (<20 ng/ml) was observed in 251 (64,1%) out of 391patients. A liver fibrosis stage of 0–2 was observed in 321 (84,9%) out of 378 patients, while 57 (15%) out of 378 patients showed a liver fibrosis stage of 3–4.

*Abbreviations*: BMI: body-mass-index, ALT: alanine aminotransferase, AST: aspartate aminotransferase, gGT: gamma-glutamyl-transferase, HOMA: homeostatic model assessment of insulin resistance, AP: alkaline phosphatase, TSH: thyroid stimulating hormone, IP10: interferon-γ-inducible-protein-10, HCV: hepatitis C virus.

*Missing data*: Cholesterol levels were missing in 14 patients, Baseline Vitamin D levels were missing in 7 patients, Baseline IP10 levels were missing in 7 patients, Baseline viral load values were missing in 16 patients, Liver biopsy status was missing in 20 patients. HOMA-index values were missing in 74 patients, Triglyceride levels were missing in 15 patients, AP levels were missing in 4 patients, Ferritin levels were missing in 10 patients, Vitamin D levels on week 24 were missing in 69 patients, IP10 levels on week 1 were missing in 23 patients, IP10 levels on week 4 were missing in 42 patients, TSH values were missing in 6 patients, Genotype of the *IL28B*-gene was missing in 31 patients, Genotype of the *CYP27B1*-gene was missing in 32 patients, Genotype of the *CYP2R1*-gene was missing in 32 patients, Genotype of the *DHCR7*-genes was missing in 24 patients, Genotype of the *CYP24A1*-gene was missing in 24 patients, Genotype of the *DBP*-genes was missing in 24 patients.

### Baseline and under Therapy Vitamin D Levels Unable to Predict Treatment Outcome

**Figure 1:**
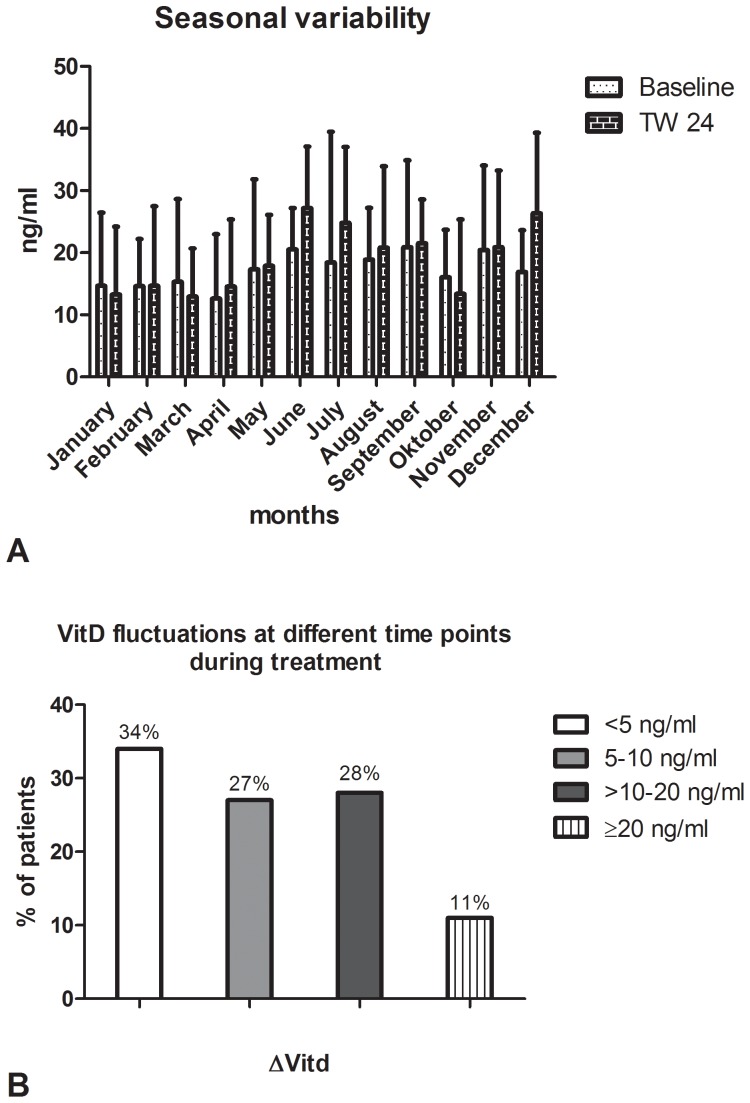
VitD concentrations vary dependent on the month of sample obtainment (1A), whereas fluctuations between baseline-VitD and TW24-VitD-values (ΔVitd) are observed as well (1B).

25(OH)D_3_- levels have been assessed retrospectively prior to and during antiviral treatment (on therapy week 24, TW24) in respectively available blood samples. Mean concentrations at both time points (18,7 ng/ml and 19,5 ng/ml respectively) reveal a VitD-deficiency among the patient cohort (Table 1), since levels >30 ng/ml are proposed as normal VitD concentrations in serum by clinical guideline recommendations [19]. In our analysis we further observed variations within VitD-levels during the course of antiviral therapy, whereas environmental factors like the month of blood sample collection, affected significantly the levels of the measured 25(OH)D_3_-concentrations (Figure 1A). Furthermore, during the long-lasting course of antiviral therapy 66% of patients exhibited a VitD-level-difference (ΔVitD) of ≥5 ng/ml between VitD-levels assessed at baseline and at TW24 (Figure 1B). Impressively in 39% of patients even more pronounced variations of VitD-levels (>10 ng/ml) have been observed (Figure 1B). However, no significant association between VitD-concentrations and SVR-rates was observed in uni- and multivariate analysis for both time points (Table 2). Furthermore, no significant association of baseline VitD levels to HCV-RNA kinetics was observed. Patients with rapid viral response (RVR) showed a positive, however statistically non-significant correlation to higher baseline VitD values as compared to patients with no RVR (21,04 versus 18,84 ng/ml respectively, *P* = 0.554). Also ΔVitD-values were despite the observed variability unable to predict SVR rates (*P* = 0.058). When solely patients with severe VitD-deficiency (<10 ng/ml) or normal serum VitD-values (≥30 ng/ml) at baseline were included in the analysis, SVR-rates of 61% versus 48% were observed, respectively. Patients with a severe VitD-deficiency both at baseline and TW24 achieved a SVR in 74% while patients with normal VitD-concentrations both at baseline and TW24 achieved a SVR in 76% of cases with no significant differences observed in both groups**.** When serum probes were taken during the summer months (May to August) mean VitD levels were slightly higher as compared to serum probes taken during the winter months (November to February) (18,9 versus 16,4 ng/ml respectively). However, no significant associations of VitD levels to SVR rates were observed both when only patients with VitD levels evaluated during the summer (16,2 versus 21,2 for SVR versus nonSVR respectively, *P* = 0.056) as well as when only patients evaluated during the winter months were considered in the analysis (16,3 versus 17,3 for SVR versus nonSVR respectively, *P* = 0.8).

**Table 2:** Uni- and multivariate analysis of predictors of SVR to antiviral therapy.

Patient characteristics	Univariate analysis			Multivariate analysis	
	SVR Mean (Range)	non-SVR Mean (Range)	p-value	OR (95% CI)	p-value
**Age (years)**	42 (18–68)	46 (19–70)	<0.001	1.029 (1.002–1.057)	0.032
Sex (female/male) *n, [%]	106* [26,6%]/111*[27,8%]	76* [19,0%]/104* [26,1%]	0.1		
**BMI (kg/m^2^)**	24.7 (17.3–45.5)	24.7 (16.6–42.9)	0.5		
**ALT_fact (times upper limit** **of normal, IU/l)**	1.64 (0.31–9.8)	1.56 (0.36–12.8)	0.4		
**AST_fact (times upper limit** **of normal, IU/l)**	1.09 (0.43–7.96)	1.1 (0.48–7.89)	0.3		
**GGT_fact (times upper limit** **of normal, IU/l)**	0.71 (0.15–7.31)	1.28 (0.25–16.08)	<0.001	1.46 (1.068–1.995)	0.017
**HOMA-index**	1.75 (0.3–20)	2.55 (0.1–22.6)	<0.001	0.949 (0.87–1.036)	0.2
**Cholesterol (mg/dl)**	182 (101–320)	166.2 (61–289)	<0.001	0.983 (0.975–0.991)	<0.001
**Triglyceride (mg/dl)**	89 (25–422)	93 (16–490)	0.7		
**AP (IU/l)**	65 (32–181)	71.5 (32–204)	<0.001	1.011 (0.997–1.026)	0.09
**Ferritin (µg/l)**	101 (3.7–780)	153 (6–1846)	<0.001	1.002 (1.000–1.004)	0.032
**Vitamin D baseline (ng/ml)**	15.8 (3–76.2)	17.6 (3.1–84.3)	0.09		
**Vitamin D TW 24 (ng/ml)**	16.7 (3.7–56.9)	16.35 (3.2–61.9)	0.5		
**IP10 baseline (pg/ml)**	228 (58.4–2000)	279 (41.2–2000)	0.009	1.000 (0.999–1.001)	0.2
**TSH (µIU/ml)**	1.37 (0.02–12)	1.24 (0.01–5.03)	0.2		
***IL28B-rs12979860*** ** (CC/non_CC)**	85 [23,1%]/32 [8,79%]	118 [32,1%]/132 [35,9%]	<0.001	2.476 (1.289–4.757)	0.006
***CYP27B1-rs10877012*** **(AA/non_AA)**	27 [7,3%]/176 [48,0%]	12 [3,29%]/151 [41,2%]	0.06		
***CYP2R1- rs10741657*** **(AA/non_AA)**	33 [9,0%]/170 [46,4%]	18 [4,9%]/145 [39,6%]	0.1		
***DHCR7-rs12785878*** **(TT/non_TT)**	100 [26,6%]/109 [29%]	98 [26,1%]/68 [18,1%]	0.031	0.745 (0.417–1.331)	0.3
***CYP24A1-rs6013897*** **(TT/non_TT)**	128 [34,1%]/81 [21,6%]	104 [27,7%]/62 [16,5%]	0.7		
***DHCR7-rs7944926*** **(GG/non_GG)**	106 [28,2%]/103 [27,4%]	98 [26,1%]/68 [18,1%]	0.1		
***DBP-rs2282679*** **(TT/non_TT)**	111 [29,6%]/98 [26], [1]	85 [22,6%]/81 [21,6%]	0.7		
***DBP-rs4588*** **(CC/non_CC)**	107 [28,5%]/102 [27,2%]	81 [21,6%]/85 [22,6%]	0.6		
***DBP-rs7041*** **(GG/non_GG)**	65 [17,3%]/144 [38,4%]	50 [13,3%]/116 [30,9%]	0.8		
**Fibrosis stage (0–2/3–4)**	184 [48,6%]/19 [5,0%]	137 [43,0%]/38 [10,0%]	<0.001	1.489 (0.562–3.946)	0.4

Only patients with complete data for the remaining covariates (277 out of 398) and with significant variations in the univariate analysis were included in multivariate analyses. Missing data and abbreviations are illustrated in the legend of Table 1. A VitD deficiency (<20 ng/ml) was observed in 251 (64,1%) out of 391patients. A liver fibrosis stage of 0–2 was observed in 321 (84,9%) out of 378 patients, while 57 (15%) out of 378 patients showed a liver fibrosis stage of 3–4.

### SNP’s within Vitamin D Regulating Enzymes

Various SNP’s within several enzymes of VitD metabolism (*CYP2R1, CYP27B1, CYP24A1* and *DHCR7*) have been by previous studies identified to have substantial impact on the serum concentrations of 25(OH)D_3_ [28]_._ In our analysis no significant correlation of most SNP’s to SVR rates was identified (Table 2). In particular, we evaluated the predictive potential of the already proposed *CYP27B1*-SNP (rs10877012) in the promoter of the VitD-1α-Hydroxylase [22] on treatment outcome in our patient cohort. Regarding the induction of SVR we observed solely a non-significant trend of the *AA*-allele in univariate analysis (*P* = 0.067) (Table 2). The only significant association to SVR rates was observed for the *DHCR7*-SNP (rs12785878) in univariate analysis (*P* = 0.031), whereas adjustment for further predictive factors eliminated this parameter from the regression model in multivariate analysis (Table 2). Further SNP’s within VitD binding proteins (*DBP*-rs4588 and -rs7041) previously highlighted to associate significantly with the outcome of antiviral therapy [12] didn’t show any significant correlation to SVR rates in our patient cohort (Table 2). Furthermore, solely *DHCR7*-SNP’s (rs12785878 and rs7944926) showed a significant association to baseline VitD levels (*P* = 0.014 and *P* = 0.002 respectively) (Table 3) whereas ΔVitD-values during antiviral therapy didn’t show any significant association to all SNP’s analyzed (Table 3).

**Table 3:** SNP’s within VitD regulating enzymes in association with baseline VitD values and VitD variations (ΔVitd) upon antiviral therapy.

Polymorphism	VitD <20 ng/ml	VitD ≥20 ng/ml	p-value	ΔvitD <0 ng/ml	ΔvitD ≥0 ng/ml	p-value
**CYP27B1-rs10877012** **(AA/nonAA)^1^**	28/205	11/122	0.2	16/151	21/141	0.3
**CYP2R1- rs10741657 (AA/nonAA)^2^**	30/203	21/112	0.4	23/144	18/144	0.4
**DHCR7-rs12785878 (TT vs nonTT)^3^**	113/120	81/50	0.014	78/75	86/69	0.4
**CYP24A1-rs6013897 (TT vs nonTT)^4^**	138/95	84/47	0.3	9/144	5/150	0.2
**DHCR7-rs7944926 (GG vs nonGG)^5^**	114/119	86/45	0.002	81/72	88/67	0.4
**DBP-rs2282679 (TT vs nonTT)^6^**	119/114	68/61	0.7	81/72	76/79	0.4
**DBP-rs4588 (CC vs nonCC)^7^**	112/121	67/64	0.5	77/76	70/85	0.3
**DBP-rs7041 (GG vs nonGG)^8^**	68/165	43/89	0.4	48/105	46/109	0.7

1,2 ΔVitd values were missing in 37 patients, ^3–8^ΔVitd values were missing in 66 patients.

### Parameters Independently Predicting SVR-rates

**Figure 2:**
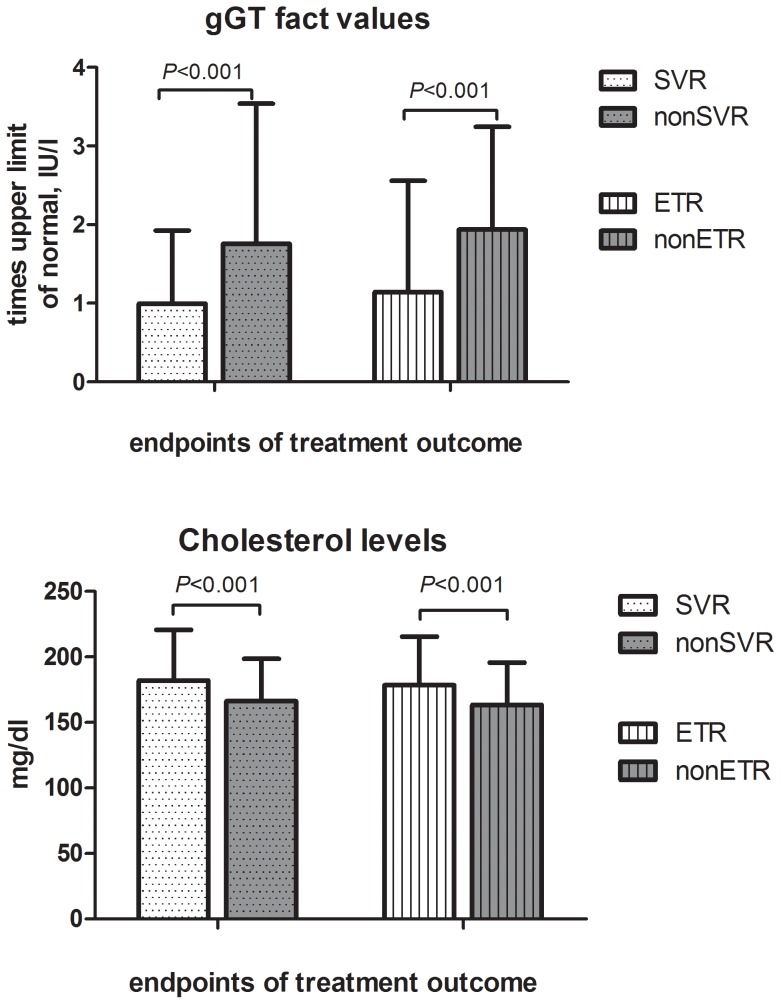
High cholesterol- and low gGT- levels associate with achievement of SVR and ETR.

**Figure 3:**
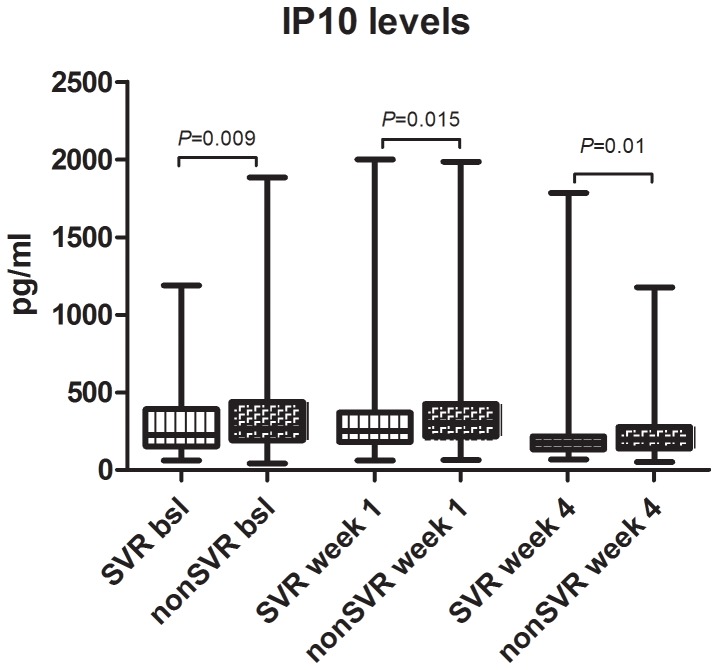
Variations of IP10-levels in patients with and without SVR at baseline, week 1 and 4 upon treatment initiation.

Several parameters were significantly associated with the induction of SVR in univariate analysis as shown in Table 2. However, only a part of these parameters constituted independent predictors of treatment response, identified by a multivariate logistic regression model, in our analysis. The induction of SVR was independently predicted by age (OR = 1.029, CI = 1.002–1.057, *P* = 0.032), cholesterol (OR = 0.983, CI = 0.975–0.991, *P*<0.001), ferritin (OR = 1.002, CI = 1.000–1.004, *P* = 0.032), gGT (OR = 1.467, CI = 1.068–1.995, *P* = 0.017) and *IL28B*-genotype (OR = 2.476, CI = 1.289–4.757, *P* = 0.006). Low baseline gGT-levels and high baseline cholesterol concentrations feature the two clinical parameters with the highest independent predictive potential beside demographic and genetic patient characteristics (Figure 2). Low IP10-levels at baseline, therapy week 1 and therapy week 4 were significantly associated with the induction of SVR (*P* = 0.009, *P* = 0.015, *P* = 0.010 respectively) in univariate analysis, whereas no significant associations were observed in multivariate analysis (Table 2). 358 out of 391 patients had an IP10 baseline level of <600pg/ml which was suggested by previous studies to be favorable for the achievement of SVR upon PEG-IFN/RBV combination therapy [29]. The observed variations of IP10 levels during the course of antiviral therapy (Figure 3) were unable to predict treatment outcome (data not shown). Since many studies suggested a significant improvement in the prediction of SVR by combination of IP10-levels and *IL28B*-genotype [29], [30] we also analyzed the predictive ability of IP10-levels separately in patients with the favorable *IL28B*-CC-genotype and in non-CC-patients. Accordingly, in *IL28B*-CC-patients achieving an SVR, IP10-levels were lower (243 pg/ml) than in patients without SVR (265 pg/ml) but not significantly associated with SVR (*P* = 0.7). However, in *IL28B*-nonCC-patients a significant association between IP10-levels and SVR was observed (222 and 299 pg/ml, in SVR and nonSVR-patients respectively, P = 0.002).

## Discussion

In the present study we examined thoroughly the predictive potential of baseline and under therapy VitD-levels as well as of their fluctuations during antiviral therapy on treatment outcome in a well characterized cohort of 398 genotype 1 patients treated prospectively with PEG-IFN/RBV for variable time periods. Furthermore, we included a comprehensive evaluation of genetic polymorphisms within VitD regulating enzymes that have been previously shown to affect VitD concentrations or to associate with outcome of antiviral therapy. Among all parameters screened we identified younger age, elevated baseline cholesterol levels, low baseline gGT and ferritin levels as well as the *IL28B*-CC-genotype as independent predictors of reaching a SVR whereas VitD levels, their under-therapy-fluctuations and most of the SNP’s within VitD regulating enzymes were not able to predict treatment outcome.

Recent findings from various studies investigating the role of VitD in chronic HCV infection offer conflicting evidence regarding the predictive potential of pretreatment VitD concentrations upon antiviral therapy regimes. On the one hand low pretreatment VitD-concentrations are suggested to correlate with poor responsiveness to antiviral therapy [10]–[14] and on the other hand in 269 patients infected with various HCV genotypes [15] and in 317 patients infected with genotype 1 [22], all treated prospectively with PEG-IFN/RBV, VitD levels seem unable to predict treatment response. Furthermore, baseline VitD concentrations failed to prognosticate outcome of antiviral therapy in HCV/HIV-coinfected patients as well [16], [17], thus further subsidizing the already observed controversy. However, assessment of VitD concentrations solely prior to treatment initiation as well as incomplete consideration of environmental and genetic factors affecting VitD levels constitute possible sources of bias in hitherto existing analyses. In the present study we intended to overcome some of these limitations by measuring VitD concentrations also at TW24 and by considering the seasonal variability of VitD levels and the effect of SNP’s within major regulatory enzymes. Since HCV infection has been suggested by previous studies to co-associate with VitD-deficiency [22], [31], [32], we further intended to examine additionally whether the eradication of HCV had a reciprocal effect on VitD levels. Moreover, it remains unclear so far whether the additional evaluation of VitD levels during antiviral therapy plays a role in predicting SVR rates. Thereby we evaluated VitD concentrations at TW24 and beside significant variations within VitD concentrations neither associations to therapy outcome nor normalization of VitD levels in patients with SVR were identified at TW24.

**Figure 4:**
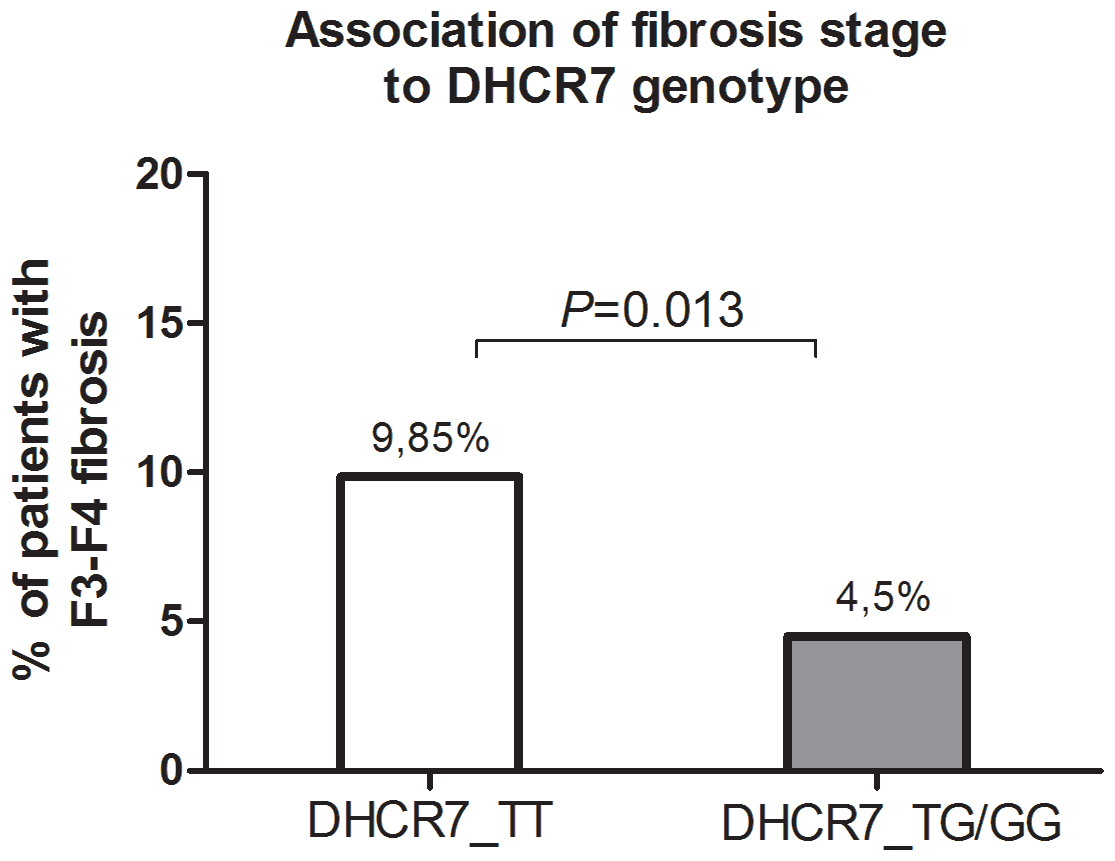
The *DHCR7-TT-*genotype of the *rs12785878* SNP shows a significant association to stage of fibrosis.

Our results reveal substantial fluctuations of 25(OH)D_3_ levels during antiviral therapy, which are affected by the month of blood collection, yet no association to treatment outcome was identifiable. While patients achieving a SVR feature a slight elevation of VitD levels between baseline and TW24 (from 15,8 ng/ml to 16,7 ng/ml) with nonSVR-patients featuring a slight reduction respectively (from 17,6 ng/ml to 16,3 ng/ml), no statistical significance has been observed between both groups. Also the analysis of an already identified SNP (rs10741657) [33] within the *CYP2R1* enzyme, with the latter being expressed in the liver and therefore among others being responsible for the generation of 25(OH)D_3_, showed no significant association to treatment outcome. In contrast, the also already proposed SNP within the *CYP27B1* enzyme (rs10877012), responsible for the generation of the active 1,25(OH)_2_D_3_, showed a non-significant trend to SVR- (Table 2) in univariate analysis. We assume, that we were unable to confirm the observed significant association [22] due to the fact that the amount of patients being homozygote for the AA-allele was very limited (39 out of 366) in our patient cohort. Despite the fact that according to a recent study a prediction of SVR was possible depending on identification of the wild type allele of the VitD binding protein (*DBP*-rs4588 and -rs7041) [12], no significant associations have been identified within our patient cohort. However, we identified a significant association between SVR rates and the rs12785878 SNP within the 7-dehydrocholesterol reductase (*DHCR7*). *DHCR7* converts 7-dehydrocholesterol to cholesterol thus removing the substrate from the synthetic pathway of VitD and finally regulating VitD levels [28], which is in accordance with our current observations (Table 3). Furthermore, two recent studies identified the rs12785878 SNP within *DHCR7* to associate significantly with liver fibrosis progression in genotype 1 HCV infected patients [34] and with HCV-associated hepatocarcinogenesis respectively [35]. Thus, we further examined whether the rs12785878 SNP within DHCR7 associated with fibrosis progression in our patient cohort as well. Thereby, we observed a significant association of the TT-genotype to fibrosis stage F3–F4 (*P* = 0.013) (Figure 4), which is in accordance with previous published results [34]. Since patients with low fibrosis show higher responsiveness to antiviral therapy compared to patients with advanced fibrosis or cirrhosis, the significant association of the *DHCR7* genetic polymorphism to SVR rates observed in our study appears logically consistent and adds further valuable information regarding the role of *DHCR7* and VitD metabolism in chronic HCV infection.

While clinical guidelines define VitD levels of ≥30 ng/ml as normal [19], we observed an overall VitD deficiency within the entire patient cohort which persisted even during antiviral therapy (Table 1). The already mentioned previous studies suggesting VitD values being predictive of treatment response included patients with overall higher mean levels of 25(OH)D_3_ concentrations [10], [12], [13]. Thus, our results imply that the predictive ability of baseline 25(OH)D_3_ values observed in previous studies disappears in deficient concentration ranges. A possible explanation of this observed phenomenon is that the proposed synergistic effect of 1,25(OH)_2_D_3_ and IFNα on the IFN-gene-expression and HCV replication [15] may be so substantially diminished at deficient concentration ranges that the predictive potential of VitD concentrations disappears. Furthermore, it is well known that 25(OH)D_3_ serum concentrations correlate poorly with serum and tissue levels of the bioactive vitamin D metabolite calcitriol [18]. In a sub-analysis including only patients with normal VitD-concentrations (≥30 ng/ml) still no significant association was observed between baseline VitD-levels and SVR rates (*P* = 0.1). However, this sub-analysis could be confounded by the small amount of patients having normal VitD-levels at baseline (n = 48) within our patient cohort. Moreover, as above mentioned, primary studies observing a positive correlation of VitD levels to SVR rates were conducted in countries with higher sun exposure and thus higher baseline VitD levels [11]–[14]. However, further studies with HIV/HCV-coinfected patients, which were conducted in countries with high sun exposure as well, failed to show a positive correlation of VitD levels and SVR rates [16], [17], with a recent swiss cohort [15] even showing a positive trend between lower VitD levels and higher SVR rates, which is in line with our current findings. Since all trials, both in the Mediterranean area as well as in Northern Europe, showed similar SVR rates, we may conclude that variations within VitD levels are unable to predict SVR rates especially in regions with higher rates of VitD-deficiency as this is the case for Northern Europe.

Regarding the role of IP10, previous studies showed a significant decline during antiviral therapy which remains consistent in responders after HCV eradication and resolves with persistence of chronic HCV infection in non-responders [36], [37]. We observed a slight increase of IP10 levels at week 1 and a consecutive decrease of IP10-levels at week 4, with IP10 levels remaining predicting of SVR at each time point assessed, thus underlying the importance of IP10 in predicting treatment outcome. Therefore, we propose that especially the comprehensive evaluation of IP10 levels during antiviral therapy, as for instance at week 4, may increase the predictive potential of viral kinetics assessed at this time point of antiviral therapy. Moreover, low baseline gGT- and elevated cholesterol-levels appear as the strongest predictors of SVR after the *IL28B*-CC-genotype in our analysis. Since both parameters are routinely assessed in clinical biochemical screenings the specific consideration of both factors in clinical decisions might offer valuable comprehensive information regarding treatment prediction according to our analysis. A subanalysis within our patient cohort including patients with IL28B-CC-genotype, low IP10-levels (<600ng/ml) [29], high cholesterol-levels (cut-off value of 174 mg/ml, calculated by ROC-analysis) and non-elevated gGT-values (n = 41), showed an SVR-rate of 78,1% for these patients, which is similar to SVR-rates achieved by the currently standard-of-care triple therapy in genotype 1 patients with PEG-IFN/RBV in combination with telaprevir or boceprevir, both direct ketoamide inhibitors of the HCV-NS3-protease. Thus, we may conclude, that the PEG-IFN/RBV combination therapy could still display an option for carefully preselected patients according to the above mentioned treatment predictors especially in areas with limited health care budget.

Although offering promising results, our study had some limitations. Beside the fact that it is retrospective, VitD concentrations and SNP’s were available only in a subgroup of the entire patient cohort with possible selection bias not being excludable. With an overall VitD-insufficiency observed in the patient cohort, our study provides information regarding responsiveness to antiviral treatment in VitD-insufficient genotype 1 HCV patients. Despite our effort to minimize confounding factors such as consideration of seasonal and on-treatment variability (Figure 1), data on prevalence of osteoporosis and parathyroid dysfunction were lacking in our analysis, thus potentially affecting the results of our studies. Furthermore, our study was conducted prior to the approval of direct acting NS3 protease inhibitors and therefore it does not resemble the current standard of care for genotype 1 HCV infected patients. However, in many countries worldwide no direct antiviral agents are yet available with PEG-IFN/RBV being still the standard of care.

In conclusion, our study implies that VitD concentrations are not a reliable prediction parameter in genotype 1 HCV patients undergoing PEG-IFN/RBV antiviral therapy. Fluctuations in VitD levels during antiviral therapy may be due to environmental factors rather than to virus- or antiviral therapy-related effects whereas the evaluation of SNP’s within VitD regulating enzymes constitutes an important component in further deciphering the role of VitD metabolism in chronic HCV infection.

## Conclusions

- Vitamin D levels assessed at baseline and at therapy week 24 show substantial variations during antiviral therapy but are unable to predict treatment outcome.
- The rs12785878 SNP within *DHCR7* is significantly associated with sustained viral response rates, whereas further SNP’s within the Vitamin D binding protein and Vitamin D regulating enzymes showed no significant associations.
- Baseline and under therapy IP10-levels are independently predicting therapy outcome especially in IL28B-nonCC-patients while low baseline gGT- and elevated cholesterol-levels appear as the strongest predictors of therapy outcome after the IL28B-CC-genotype.
